# Preparation, physicochemical properties, and *in vitro* digestion characteristics of coconut diacylglycerol oil

**DOI:** 10.1016/j.fochx.2025.102991

**Published:** 2025-09-10

**Authors:** Jiao-jiao Yin, Xin-feng Li, Shu Wang, He Huang, Zhuo-long Guan, Xing-he Zhang, Wu Zhong, Pan Gao, Dong-ping He

**Affiliations:** a Key Laboratory of Edible Oil Quality and Safety for State Market Regulation, Wuhan Polytechnic University, Wuhan 430023, China; b Key Laboratory for Deep Processing of Major Grain and Oil of Ministry of Education in China, College of Food Science and Engineering, Wuhan Polytechnic University, Wuhan 430023, China; c Wuhan Institute for Food and Cosmetic Control, 1137 Jinshan Avenue, Wuhan 430040, China

**Keywords:** Coconut oil, Coconut diacylglycerol oil, Enzymatic transesterification, Physicochemical properties, *In vitro* digestion

## Abstract

Coconut oil (CO), rich in medium-chain fatty acids and lauric acid, has gained popularity for its functional benefits. This study focused on the preparation and analysis of coconut diacylglycerol oil (CO-DAG), exploring its physicochemical properties and *in vitro* digestion characteristics, providing a theoretical basis for its application in functional oil. Through enzymatic transesterification using Novozym 435, CO-DAG with a DAG content of 54.65 % was obtained under optimal conditions (substrate ratio 1:2.5, reaction time 9.18 h, temperature 65 °C, enzyme dosage 3.60 %). Compared to CO, CO-DAG exhibited a higher acid value and anisidine value but a lower peroxide value. Additionally, CO-DAG demonstrated a reduced lauric acid content. Hazardous substances levels were within acceptable ranges. *In vitro* digestion studies revealed that CO-DAG was more stable during digestion, and exhibited an 11.2 % higher free fatty acid release than CO. These findings propose a novel approach for the advanced processing and enhanced utilization of CO.

## Highlights

• Developed a CO-DAG with high DAG content (54.65 %) *via* enzymatic methods.
• The lauric acid content in the oil was changed by enzymatic transesterification.
• CO-DAG shows faster digestion kinetics and 11.2 % more FFA release than CO.

## 1 Introduction

Coconut oil (CO), as a novel edible oil, has gained significant popularity in the market in recent years. It primarily consists of medium-chain fatty acid (MCFA) glycerides, with lauric acid content as high as 41 % - 56 % (Marina et al., 2009). MCFA are easily digestible and absorbable, and can rapidly supply energy. Lauric acid possesses bioactivities such as antibacterial and antiviral properties. Thus, CO has demonstrated high application value in aspects such as medicine, weight loss and beauty, regulation of human metabolism, and plasma blood lipids, and is regarded as a popular functional oil (Rohman et al., 2019). Beyond its inherent functional properties, coconut oil can also be modified to produce other functional lipids, which further extend its potential applications.

Among functional lipids, diacylglycerol (DAG), particularly 1,3-DAG, has recently garnered considerable attention for its diverse physiological effects. These include reducing visceral fat accumulation, inhibiting body weight gain, and lowering serum triglyceride (TAG) level (Rudkowska et al., 2005), thus offering multiple functional benefits in the context of metabolism health. Furthermore, previous study has demonstrated that coconut DAG oil (CO-DAG) exhibits a more pronounced effect on enhancing body fat metabolism compared to long-chain peanut DAG oil (Lu et al., 2020). In addition to its functional properties, CO-DAG finds wide application in the food industry. Its excellent emulsifying, rheological, thermodynamic properties make it promising for use in dairy products, coffee, and various other food sectors (Liao et al., 2025; Liu, Xu, et al., 2023).

The synthesis of diacylglycerol (DAG) is predominantly facilitated through enzymatic catalysis, which offers several advantages over chemical catalysis, including mild reaction conditions, high specificity, and reduced production of toxic byproducts, thus aligning with the principles of green manufacturing (Shi et al., 2024). However, the economic cost associated with enzymatic catalysis is significant. To mitigate these costs, immobilized enzymes are frequently employed due to their ease of recovery and reuse. Furthermore, immobilized enzymes exhibit enhanced resistance to variations in pH, temperature, and organic solvents compared to their free counterparts (Almeida et al., 2020). Despite extensive research on DAG preparation, there is a paucity of studies focusing on the preparation of CO-DAG. For instance, some researchers have utilized immobilized enzyme technology and enzymatic transesterification to produce CO-DAG oil, achieving a DAG content of 30 % (Almeida et al., 2020). Nevertheless, given that the DAG content remains below 40 %, further investigation into CO-DAG preparation methods is necessary to fulfill industrial production requirements.

In addition to the basic physicochemical properties, the digestion characteristics of lipids play a vital role in reflecting their quality and impact on health. Variations in fatty acid position, carbon chain length, double bond number, or double bond position lead to diverse chemical and physical properties among different lipid types, thereby influencing their digestion characteristics in the body. In contrast to complex, expensive, time-consuming, and ethically challenging *in vivo* tests, *in vitro* digestion serves as a commonly employed method to assess the digestive behavior of oils (Diao et al., 2020). Recently, the utilization of INFOGEST standardized *in vitro* digestion method (static) and pH-stat digestion methods (dynamic) are widely used in the lipid digestion. The INFOGEST method is highly standardized, accurately simulating the human gastrointestinal digestion environment, thereby ensuring excellent comparability and reproducibility of experimental outcomes. In contrast, the pH-stat method enables real-time monitoring of enzyme hydrolysis reactions, offering continuous hydrolysis kinetic data. Compared to TAG oil such as lard and sesame oil, the corresponding DAG exhibit a higher digestion rate and greater fatty acid release capacity during *in vitro* simulated digestion (Diao et al., 2020; Liu, Li, et al., 2021). However, up to now, there is still a lack study about the digestion characteristics of CO-DAG.

Considering the factors mentioned above, the study initially determined the optimal preparation conditions for CO-DAG through single-factor experiments and response surface methodology. Subsequently, a comparative analysis was performed to evaluate the fundamental physicochemical properties and hazardous compounds present in conventional CO, the prepared CO, and the optimized CO-DAG. Furthermore, an *in vitro* digestion model was developed to assess the rate and extent of free fatty acids (FFAs) released from CO and CO-DAG. Additionally, the particle characteristics, including size, charge, and microstructure, during the digestion of CO and CO-DAG under simulated gastrointestinal conditions were examined. The objective of this study is to enhance the high-value application of CO and lay the foundation for the potential utilization of CO-DAG in the production of functional lipids.

## 2 Materials and methods

### 2.1 Materials

The commercially available coconut oil (AC-O) was purchased from the market. CO obtained from Wenye 4 (W4) coconut variety using cold-pressed (W4—C) and hot-pressed (W4—H) methods was referenced from our previous study (Guo et al., 2025). The lipases Lipozyme TL IM, Novozym 435, and Lipozyme RM IM were purchased from Novozymes A/S (Bagsvaerd, Denmark). High-performance liquid chromatography grade n-Hexane, methanol, and isopropanol were purchased from Macklin Biochemical Technology Co., Ltd. (Shanghai, China). Reagents used in this study purchased from Sinopharm Chemical Reagent Co., Ltd. (Shanghai China), and were of analytical grade.

### 2.2 Preparation of CO-DAG oil

In this study, W4—C oil, which exhibits optimal quality in our previous investigation (Guo et al., 2025), was selected as the raw material for the transesterification reaction with monoglycerides to synthesize DAG. Lipase, recognized for its efficacy as a biocatalyst in DAG preparation, is available from a variety of sources. For this study, three commercially available immobilized lipases commonly employed in DAG synthesis, were selected: Novozym 435 (derived from *Candida antarctica*), Lipozyme TL IM (derived from *Thermomyces lanuginosus*), and Lipozyme RM IM (derived from *Rhizomucor miehei*). With reference to the enzymatic transesterification method for DAG synthesis, 6.6425 g (0.01 mol) of CO was precisely weighed and mixed with monoglycerides as reaction substrates. Lipase was subsequently added, and the mixture was incubated in a thermostatic water bath shaker at 200 r/min. After the reaction was completed, the oil mixture was centrifuged at 5000 r/min for 10 min. The supernatant was collected and filtered to obtain the CO-DAG.

The contents of DAG were analyzed using HPLC with the following parameters: chromatographic column: Aglient ZORBAX SB C18 column (100 mm × 2.1 mm, 1.8 μm); mobile phase: n-hexane: isopropanol: methanol (15:1:0.003, *v*/v); flow rate: 25.0 mL/min; column temperature: 30.0 °C; injection volume: 10.0 μL.

### 2.3 Experimental design for response surface methodology (RSM)

Based on the results of single-factor experiments, a three-level four-factor Box-Behnken design was used to evaluate the interactive effects of reaction variables on the DAG content in CO-DAG. The factors used included substrate ratio, reaction time, reaction temperature, and enzyme dosage, details are shown in Appendix Table S1.

### 2.4 Determination of physicochemical index

The acid value (AV), peroxide value (PV) and anisidine value (*p*-AV) were determined according to the method specified in our previous study (Guo et al., 2025). The total oxidation value (TOTOX) comprehensively characterizes the oxidation process of oils and fats. The calculation formula of TOTOX is as follows:

(1) TOTOX=2×PV+p−AV

### 2.5 Determination of fatty acid composition

The profile of fatty acids was analyzed utilizing an Agilent 7890 A gas chromatograph (GC) system (Palo Alto, CA, USA), following the methodology outlined in our earlier research (Guo et al., 2025). Briefly, fatty acid methyl esters (FAMEs) were generated by dissolving 0.20 g of the oil sample in 2.0 mL n-hexane and adding 200 μL of a potassium hydroxide-methanol solution. Analysis was performed using an Agilent 7890 A GC system equipped with a Trace TR-FAME column (60 m × 0.25 mm, 0.25 μm). Nitrogen was used as the carrier gas at 1 mL/min, with a split ratio of 100:1. The detector and injection port were set at 250 °C, and the injection volume was 1.0 μL. The GC oven temperature program involved holding at 60 °C for 3 min, ramping to 170 °C at 5 °C/min for 15 min, then to 220 °C at 2 °C/min for 10 min. FAMEs were identified *via* retention time comparison with standards and quantified as relative percentages.

### 2.6 Determination of free radical scavenging capacity

To evaluate the antioxidant activity, the DPPH radical scavenging rate was determined according to the following procedure. A 2 mL oil was mixed with 2 mL of DPPH-methanol solution and shaken thoroughly. After allowing the mixture to react in the dark for 1 h, the absorbance at 517 nm was recorded (A_s_). As a control, 2 mL of water was combined with 2 mL of DPPH-methanol, and the absorbance was measured (A_0_). Additionally, the absorbance was determined for a mixture of 2 mL of the test solution and 2 mL of methanol (A_r_). The DPPH radical scavenging rate was calculated using the specified formula (2) and the results were expressed as μmol Trolox equivalents (TE)/100 g oil.

(2) $$R\%=\left(1-\frac{A_{s}-A_{r}}{A_{0}}\right)\times 100$$

### 2.7 Determination of hazardous substances

#### 2.7.1 Determination of 3-monochloropropane-1,2-diol (3-MCPD) esters

Weigh 100 mg of the homogenized coconut oil sample and put it into a centrifuge tube. Add 500 μL of the methyl tert-butyl ether/ethyl acetate mixture (8:2 *v*/v), 250 μL of the internal standard (3-monochloropropane-1,2-diol (3-MCPD) d5, 20 μg/mL, dissolved in methyl tert-butyl ether) and 1 mL of the NaOCH_3_ solution (0.5 M). After 1 min, add 3 mL of isohexane, 100 μL of glacial acetic acid and 3 mL of the sodium chloride solution (200 g/L). Following shaking, mixing and centrifugation, carefully remove the upper liquid using a pipette. Extract the remaining aqueous layer once more with isohexane and remove the upper liquid. Subsequently, add 250 μL of the derivatization reagent (5 g of phenylboronic acid dissolved in 19 mL of acetone and 1 mL of water) to the aqueous phase. Heat it to 80 °C and keep it for 20 min, then cool it to room temperature. Extract the phenyl borate derivative of 3-MCPD with 3 mL of n-hexane. After evaporating and concentrating the extract, inject the concentrated n-hexane solution into the GC–MS system.

#### 2.7.2 Determination of glycidyl esters

Glycidyl esters was determined referred to the study of Dubois et al. (2011). Accurately weigh 0.500 g of the CO-DAG sample and dissolve it in methyl tert-butyl ether/ethyl acetate (4:1, *v*/v) to prepare a test solution with a concentration of 250 mg/mL. Activate a C18 solid-phase extraction column three times with 2 mL of methanol each time. Pipette 200 μL of the test sample solution onto the column, then elute the sample three times with 3 mL of methanol each time. Collect the eluate in a glass test tube, dry it under a nitrogen stream, add 3 mL of n-hexane/ethyl acetate (95:5, *v*/v), and vortex thoroughly to redissolve. Transfer the 3 mL of sample solution from the glass test tube to an activated Silica solid-phase extraction column. Rinse the glass test tube three times with 3 mL of n-hexane/ethyl acetate (95:5, *v*/v) each time, transfer the rinsings to the Silica column, and perform elution. Collect the eluate in a glass test tube, dry it under a nitrogen stream, add 0.5 mL of methanol, vortex thoroughly to redissolve, and finally transfer it to a liquid chromatography vial for UPLC-MS/MS analysis.

#### 2.7.3 Determination of benzo[α]pyrene

Simply, 500 mg CO was dissolved in 8 mL of n-hexane and purified using a BaP column. The column was washed with ethyl acetate/n-hexane solution (1:4 v/v) and eluted with acetone. The eluate was dried, reconstituted in 1 mL of acetonitrile, and analyzed by HPLC-FLD.

### 2.8 Establishment of an *in vitro* digestion model

The *in vitro* digestion model was developed based on methodologies outlined in previous study with some modifications (Liu, Li, et al., 2021). Initially, emulsions were formulated by homogenizing CO and CO-DAG with a bovine serum albumin (BSA) solution. The simulated saliva fluid (SSF), simulated gastric fluid (SGF), and simulated intestinal fluid (SIF) were prepared by adding α-amylase, pepsin, bile acids (10 mM) and a pancreatic enzyme mixture (100 U/mL trypsin, 200 U/mL chymotrypsin, 20 U/mL lipase), respectively.

Oral stage: The emulsion was mixed with SSF (1:1, *v*/v), and the mixture was incubated at pH 7.0 for 2 min. Gastric stage: The digestion solution after the oral stage was mixed with SGF (1:1, v/v), and the mixture was incubated at pH 2.0 for 2 h. Intestine stage: The digestion solution after the gastric stage was then diluted with SIF (1:1, v/v), and the mixture was incubated at pH 7.0 for 2 h. Throughout the entire digestion process, the temperature was kept at 37 °C.

### 2.9 Analysis of free fatty acid (FFA) release and digestion kinetics

During the intestinal digestion process, due to the production of FFA from the hydrolysis of TAG/DAG by lipase, the pH value of the digestion system decreases. The pH of the system was maintained at 7.0 *via* the addition of 0.01 mol/L NaOH. Record the volume of NaOH consumed at different time periods and calculate the release rate of FFA in the digested sample:

(3) $$\mathrm{FFA}\%=\frac{V_{\text{NaOH}}\times m_{NaOH}\times M_{Lipid}}{W_{Lipid}\times 2}\times 100$$

where, V_NaOH_ was the volume of NaOH solution consumed in the titration (L); m_NaOH_ was the molar concentration of NaOH (mol/L); M_Lipid_ was the average molar mass of the lipid (g/mol); W_Lipid_ was the total mass of the lipid initially added to the digestive system.

### 2.10 Determination of particle size and surface potential

According to the dynamic light scattering technique, the zeta potential and particle size distribution of W4—C and CO-DAG before and after digestion were analyzed using a Malvern Zetasizer Nano ZS90 zeta potential and particle size analyzer. The sample was diluted 10-fold, and 1 mL of the diluted sample was taken for measurement at 25 °C. For each sample, the measurement was carried out three times, and the average value was taken.

### 2.11 Observation of the microstructure

The microstructure of the digestion products of W4—C and CO-DAG was observed using a Nikon ECLIPSE 80i microscope. Instrument parameters: Spectral wavelength is 565 nm, excitation wavelength is 540/25 nm, and emission wavelength is 605/55 nm. The digestive solution was stained with Nile red (0.02 % Nile red dissolved in 1,2-propanediol). After mixing, 20 μL of the sample was taken to prepare a slide. After covering it with a cover glass, it was observed under a fluorescence microscope and the image was collected.

### 2.12 Data processing

Each experiment was conducted in triplicate, with results presented as the mean ± standard deviation. Data analysis was performed using one-way ANOVA *via* SPSS19.0 software. Post-hoc comparisons were made using Duncan's test, and significant differences (*P* < 0.05) were denoted by distinct letters. Graphs were generated using Origin 2021.

## 3 Results and discussion

### 3.1 Screening of lipase

**Fig. 1:**
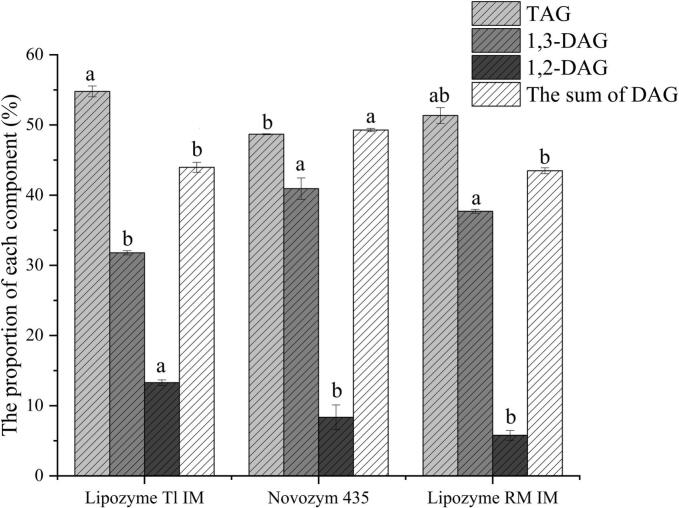
Composition percentages of individual components in oil after enzymatic interesterification. Different letters in the data plot indicate significant differences among the same components after different enzyme treatments (*P* < 0.05).

The proportions of each component in the oil after catalysis by various enzymes are shown in Fig. 1. Under the same enzyme addition amount, the proportions of DAG catalyzed by Lipozyme TL IM and Lipozyme RM IM lipases in the final preparation are 43.97 % and 43.48 %, respectively. In contrast, the total amount of DAG of Novozym 435 was 49.27 %, which was significantly higher than that of the other two groups (*P* < 0.05).

This difference is due to the highly flexible and stable structure of Novozym 435, which maintains robust activity across varying conditions and exhibits broad substrate adaptability (Ortiz et al., 2019). Conversely, Lipozyme TL IM displays 1,3-position specificity that limits substrate selectivity and reduces DAG yield (Li et al., 2015). Lipozyme RM IM, although noted for high acidolysis efficiency and magnetic reusability (Calero et al., 2014), is immobilized on an ion-exchange resin whose matrix can sterically hinder substrate access to the active site, while Novozym 435, adsorbed on a macroporous resin, exposes its active center more effectively, resulting in lower steric hindrance and higher catalytic efficiency (Duan et al., 2010).

### 3.2 Single-factor and response surface analysis

#### 3.2.1 Single-factor experiment

**Fig. 2:**
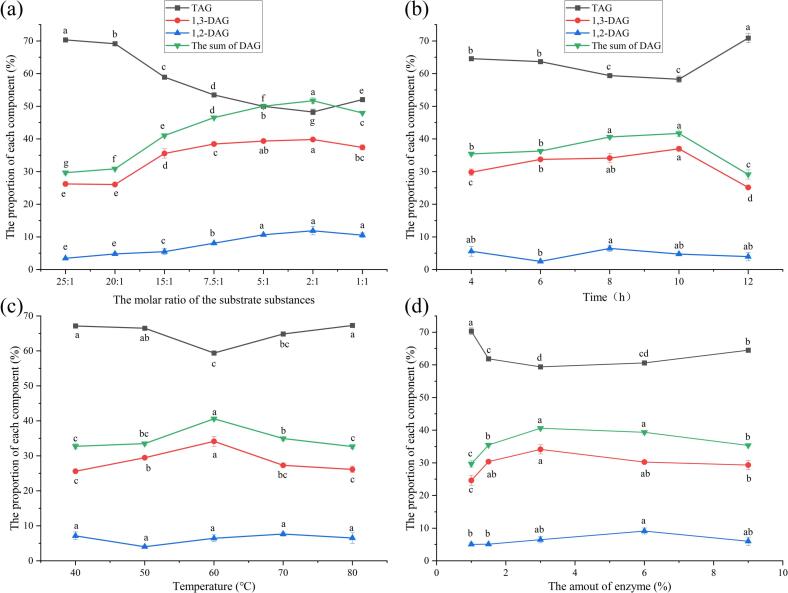
Component proportions in coconut diacylglycerol oil **at different** substrate ratios (a), reaction time (b), **reaction temperatures** (c), and the amount of enzyme (d). Different letters in the data plot indicate significant differences (*P* < 0.05).

The composition of oil under different substrate ratios is presented in Fig. 2 (a). As the substrate ratio increased, the total TAG content first decreased and then increased, whereas the DAG content exhibited the opposite trend, reaching a peak when the crude oil: monoglyceride ratio is 2:1. At this optimal ratio, the DAG yield was 50.83 %, with 1,3-DAG accounting for 40.21 % and 1,2-DAG for 10.63 %. This phenomenon can be attributed to the fact that excessive monoglyceride, as a reaction substrate, increases the viscosity of the oil, impeding the effective contact between the enzyme and substrates and consequently reducing the reaction efficiency (Chen et al., 2020). Based on the results, the optimal substrate ratio for this reaction is estimated to lie between 5:1 and 1:1.

The influence of different reaction times on the composition of oil is shown in Fig. 2 (b). As the reaction time increased, the total amount of TAG first decreased and then increased, while the content of DAG first increased and then decreased. When the reaction time is 10 h, the highest inflection point was reached, and the proportion of the produced DAG was 41.71 %. Among them, when the content of 1,3-DAG reached the highest inflection point, the proportion was 36.98 %, and the proportion of 1,2-DAG was 4.74 %. Once the substrate reaction is complete, further continuation of the enzymatic reaction will lead to the conversion of DAG into MAG and glycerol, consequently reducing the yield of DAG (Zhou et al., 2022). It can be seen from Fig. 2 (b) that the optimal reaction time may be between 8 and 12 h.

The composition of oil at different reaction temperatures is shown in Fig. 2 (c). As the reaction temperature increased, the total TAG content first decreased and then increased, while the DAG content shown the opposite trend, reaching a peak of 40.61 % at a reaction temperature of 60 °C. At this temperature, the proportion of 1,3-DAG was 34.14 %, and the proportion of 1,2-DAG was 6.47 %. Temperature is a key factor affecting enzyme activity. Insufficient reaction temperature can decrease catalytic efficiency due to reduced enzyme activity, whereas excessively high temperatures can lead to enzyme protein denaturation, thereby diminishing the effectiveness of the enzyme-catalyzed reaction (Zhou et al., 2022). It can be seen from the Fig. 2 (c) that the optimal reaction temperature may be between 50 °C and 70 °C.

The proportions of each component in the oil under different amounts of added enzyme are shown in Fig. 2 (d). As the amount of added enzyme increased, the total amount of TAG first decreased and then increased, and the content of DAG rose first and then dropped. When the total amount of added enzyme reached 3 % of the total substrate, the proportion of the produced DAG reached the highest inflection point, which was 40.61 %. Among them, the proportion of 1,3-DAG at the highest inflection point was 34.14 %, and the proportion of 1,2-DAG was 6.47 %. As a catalyst for the reaction, the enzyme does not directly participate in the reaction. However, increasing the amount of enzyme in the process can, to a certain extent, enhance the contact efficiency between the enzyme and the substrate. An excessive amount of enzyme, on the other hand, will cause the enzymes to pile up with each other, reducing the contact efficiency (Shi et al., 2024; Zhou et al., 2022). It can be seen from the Fig. 2 (d) that the optimal amount of added enzyme is likely to be between 3 % and 6 %.

#### 3.2.2 Response surface methodology

Response surface methodology is carried out based on the results of single-factor experiments. To obtain the highest DAG content, four factors are investigated, and the control table of experimental factors and levels is shown in Appendix Table S1. The experimental index Y is the total DAG content, with higher values being preferable. The Box-Behnken experimental scheme and results are presented in Appendix Table S2. Regression fitting of the table data yielded a quadratic multiple regression equation for the independent variables:

Y = 52.51 + 0.88 × A-0.022 × B + 1.88 × C + 1.13 × D + 4.26 × AB-2.36 × AC + 2.91 × AD-4.90 × BC-1.35 × BD-1.04 × CD-3.86 × A^2^–4.52B^2^–4.29C^2^–5.14D^2^.

The DAG regression analysis results are detailed in Appendix Table S3. The regression model exhibited an extremely high level of significance (*P* < 0.01), while the lack-of-fit term for Y was nonsignificant (*P* = 0.7608). The model had an R^2^ value of 0.9798 and an adjusted R^2^_Adj_ of 0.9596, indicating that the regression model is reliable, the experimental data fitted well, and the regression equation could be used to analyze and predict the optimal process conditions for generating DAG oil.

In the total DAG model, the quadratic terms A^2^, C^2^ and D^2^ had significant effects on the results (*P* < 0.05). The factors influencing DAG yield were ranked by the magnitude of F-values as follows: reaction temperature > enzyme load > substrate ratio > reaction time. Appendix Fig. S1 displays the response surface curves and contour plots of the interactive factors.

This study aims to explore the optimal process conditions for the preparation of DAG oil. Through the prediction of the regression model, the optimal process conditions were obtained as follows: the substrate ratio was 1:2.49, the reaction time was 9.18 h, the reaction temperature was 65.21 °C, and the amount of added enzyme was 3.60 %, and the proportion of DAG was predicted to be 52.92 %. During actual operation, the optimal result was slightly adjusted, that was, the material ratio of monoglyceride to crude oil added was 1:2.5, the reaction time was 9.18 h, the reaction temperature was 65 °C, and the amount of added enzyme was 3.60 %. After three verification experiments, the proportion of DAG in the prepared CO-DAG oil was 54.65 %, which exceeded the predicted value, indicating that the regression model is reliable. The DAG yield in this study is notably higher than that reported in a previous study, where enzymatic transesterification resulted in a DAG content of 30 % for the production of CO-DAG (Almeida et al., 2020). Furthermore, compared with oils rich in long-chain fatty acids, such as soybean oil, the DAG content is generally between 30 % and 50 %, in this study, the DAG content in CO-DAG is generally higher (Wang, Li, et al., 2011; Zhang et al., 2016). A possible reason is that its short carbon chain structure can significantly enhance the catalytic activity of lipase. Compared with long-chain fatty acids (such as C18:1 and C18:2 in soybean oil), medium-chain fatty acids have stronger hydrophilicity, which can enhance the interfacial contact efficiency between the substrate and the enzyme, thus increasing the conversion rate of the transesterification reaction (Marcucci et al., 2021).

### 3.3 Basic physicochemical properties

**Fig. 3:**
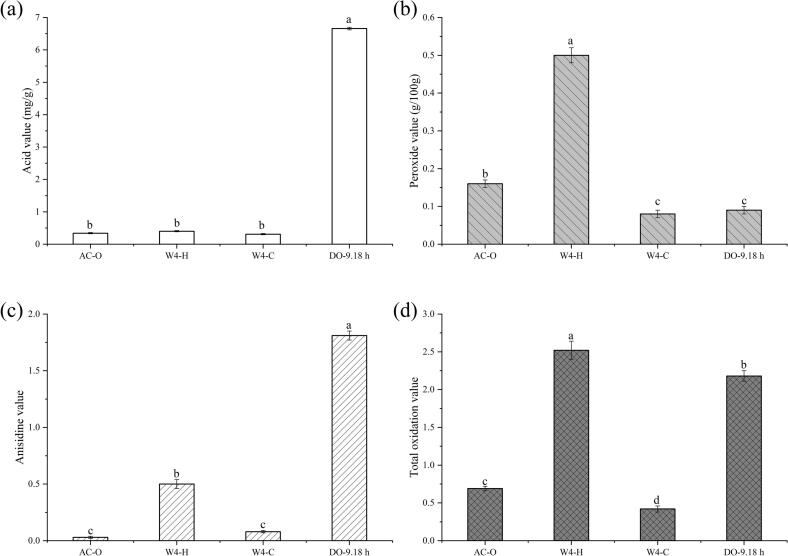
Acid value (a), peroxide value (b), anisidine value (c), and total oxidation value (d) of coconut oil and coconut diacylglycerol oil under different processing methods. Different letters in the data plot indicate significant differences (*P* < 0.05).

The acid values (AV) of CO and CO-DAG oil under different processes are shown in Fig. 3 (a). The AV of commercially available oil (AC-O) was the lowest, measured at 0.34 mg/g. To maintain stability and storge properties, AC-O typically undergoes a refining process. In contrast, the AV of W4 coconut oil prepared *via* the hot-pressed process was slightly higher at 0.40 mg/g, which may be related to the partial oxidation and decomposition of the oil caused by high-temperature pressing. Conversely, the AV of W4 coconut oil prepared by the cold-pressed process was the lowest at 0.31 mg/g, indicating its suitability for the production of high-quality oil. However, the AV of DAG oil produced by the enzymatic method generally exhibited a higher trend. This is due to the lipase-catalyzed hydrolysis of TAG during the enzymatic preparation, where the ester bonds in TAG molecules are cleaved, releasing fatty acids.

The peroxide values (PV) of CO and CO-DAG oil under different processes are shown in Fig. 3 (b). It is found that different processing methods and reaction times have a significant impact on the oxidative stability of the oil. In conjunction with the limited stipulations of the national standard GB 2716–2018, which mandates that the PV of edible vegetable oil should not exceed 0.25 g/100 g, the PV of AC-O was 0.16 g/100 g, thereby falling within the acceptable range for first-grade edible oil quality, where a PV of less than 0.10 g/100 g is considered super-grade. Conversely, the PV of CO prepared by the hot-pressed process was as high as 0.50 g/100 g, which approached the limit for industrial oil, as values exceeding 0.50 g/100 g may pose a toxicological risk. This elevation in PV is likely attributable to the high-temperature pressing process, which induces the cleavage of unsaturated fatty acid chains and the subsequent formation of hydroperoxides (Liu, Li, et al., 2023). In contrast, the cold-pressed method, characterized by its lower processing temperature, yielded a PV of merely 0.08 g/100 g. This reduction is potentially linked to the decreased activity of lipoxygenase and the inhibition of oxygen diffusion at reduced temperatures (Islam et al., 2023). Furthermore, the PV of CO-DAG decreased to 0.09 g/100 g, closely resembling that of the cold-pressed group. This low PV (< 0.10 g/100 g) highlights its suitability for the large-scale production of DAG oils.

The anisidine values (*p*-AV) of CO and CO-DAG oil under different processes are shown in Fig. 3 (c). The *p*-AV of AC-O was only 0.03, indicating that its initial content of secondary oxidation products was extremely low. The *p*-AV of hot-pressed oil (W4—H) significantly increased to 0.50, while that of cold-pressed oil (W4—C) remained at 0.08 (*p* < 0.05), indicating that high temperature exacerbates the accumulation of aldehydes during the lipid oxidation process (Soo et al., 2020). For CO-DAG oil, the *p*-AV was 1.81, which was significantly higher than that of CO treated by other methods. During the enzymatic modification of CO, some enzymes are used as catalysts, leading to a certain degree of oxidation of the oil and consequently an increase in the *p*-AV (Rocha-Uribe & Hernandez, 2008).

The total oxidation (TOTOX) values of CO and CO-DAG oil under different processes are shown in Fig. 3 (d). It integrates the comprehensive assessment of the oxidation levels of primary oxidation products (hydroperoxides) and secondary oxidation products (aldehydes), thereby quantifying the extent of damage throughout the lipid oxidation cycle. Various processing techniques and durations of enzymatic hydrolysis significantly influence the total oxidation value of the oil. Regarding process type, the TOTOX value for W4—C was the lowest at 0.42, compared to AC-O at 0.69 and W4—H at 2.52. This suggests that low temperatures effectively inhibit oxidation reactions and reduce the formation of primary (peroxides) and secondary oxidation products (aldehydes, *etc.*). Conversely, the elevated temperatures in W4—H intensified the oxidation of unsaturated fatty acids, resulting in a significant increase in the TOTOX value (Gertz et al., 2014). Among the CO-DAG, the TOTOX value of the CO-DAG group was 2.18, which, while below the risk threshold, was higher than the total oxidation value observed in W4—C. This phenomenon can be attributed to the fact that DAG oil contains a relatively higher proportion of unsaturated fatty acids. The presence of these unsaturated bonds renders them more susceptible to oxidation, thereby contributing to an elevated TOTOX. While CO also contains a certain amount of unsaturated fatty acids, the freeze-thaw process can mitigate the rate of oxidation in some extent, resulting in a lower TOTOX value compared to CO-DAG (Ng et al., 2021).

### 3.4 The fatty acid compositions of various coconut oils

The fatty acid compositions and their proportions of various coconut oils are presented in Table 1. It is known that variations in fatty acid profiles before and after the transesterification reaction are a common occurrence (Wang, Wang, et al., 2011). Similarly, in this study, the content of long-chain fatty acids (such as C16:0 and C18:0) increases, while that of medium-chain fatty acids (such as C12:0) decreases. From a mechanistic perspective, medium-chain fatty acids are relatively short, in the transesterification reaction, their smaller steric hindrance makes them more likely to detach from TAG and more readily combine with glycerol to form DAG. However, due to factors such as reaction equilibrium, their content may decrease in the overall composition. Although long-chain fatty acids exhibit larger steric hindrance and relatively lower reaction activity, the occurrence of acyl migration during the reaction process in the transesterification system provides them with more opportunities to participate in the reaction and bind to DAG, thereby leading to an increase in their content (Mao et al., 2019). It should be noted that all of the oil are in line with the characteristic of high lauric acid content in coconut oil (Xiao et al., 2018). CO-DAG rich in lauric acid has the characteristics of direct energy supply, antibacterial activity and rapid metabolism. The amphiphilic structure in the lipid structure endows it with excellent emulsification and foam stability, and the proportion of saturated fatty acids makes its oxidation stability 20 %–30 % higher than that of traditional TAG (Liao et al., 2025).

**Table 1:** The fatty acid compositions of coconut oil and coconut diacylglycerol oil.

	C6:0	C8:0	C10:0	C12:0	C14:0	C16:0	C18:0	C18:1	C18:2
AC-O	3.34± 0.03^c^	5.45± 0.08^a^	6.34± 0.00^a^	48.87± 0.11^a^	17.79± 0.03^c^	8.42± 0.01^d^	3.38± 0.02^d^	5.45± 0.01^c^	0.96± 0.00^c^
W4-H	3.03± 0.04^d^	3.93± 0.03^d^	3.84± 0.02^d^	44.45± 0.12^c^	20.85± 0.10^a^	10.99± 0.03^c^	4.57± 0.03^b^	7.18± 0.03^a^	1.16± 0.01^b^
W4-C	4.30± 0.04^b^	4.51± 0.05^b^	4.49± 0.03^b^	46.07± 0.22^b^	20.04± 0.50^b^	9.40± 0.14^b^	3.59± 0.03^c^	5.66± 0.05^b^	1.94± 0.02^a^
CO-DAG	5.50± 0.04^a^	4.40± 0.06^c^	4.24± 0.01^c^	37.43± 0.05^d^	15.43± 0.02^d^	17.09± 0.00^a^	9.71± 0.03^a^	5.08± 0.01^d^	1.12± 0.01^b^

Note: The superscript letters indicate the statistical differences at the 5 % significance level for each column.

### 3.5 Comparison of free radical scavenging ability

**Fig. 4:**
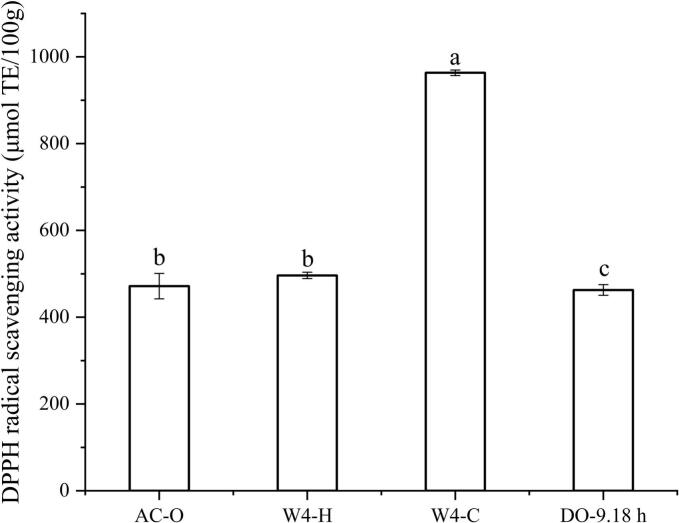
DPPH radical scavenging activity of coconut oil and coconut diacylglycerol oil under different processing methods. Different letters in the data plot indicate significant differences (*P* < 0.05).

The DPPH free radical scavenging ability of AC-O, W4—C, W4—H and CO-DAG oil with an enzymatic hydrolysis time of 9.18 h is shown in Fig. 4. The DPPH scavenging ability in CO-DAG (462.68 μmol TE/100 g), W4—H (496.33 μmol TE/100 g), and AC-O oil (471.59 μmol TE/100 g) were lower than that of CO prepared by the W4—C (963.38 μmol TE/100 g). Relevant studies have shown that CO is rich in phenolic compounds, and the free radical scavenging capacity is related to the autoxidation of phenolic substances (Liu, Chen, et al., 2021). The differences in DPPH free radical scavenging capacities among the four oils are also related to the different contents of polyphenolic substances in the oils prepared by different methods. For example, the cold-pressing method, due to its low processing temperature, can reduce the oxidative degradation of polyphenolic substances caused by high temperature, thereby retaining a higher content of polyphenols, and the corresponding DPPH free radical scavenging capacity is usually stronger (Soo et al., 2020). Meanwhile, the free radical scavenging capacity of CO-DAG prepared under the optimal process is lower than that of the other three oils. This may be due to the structural characteristics of DAG, which make the active sites of its antioxidant components more likely to be exposed to the oxidative environment during processing, thus being more susceptible to consumption and degradation, and further leading to a decrease in free radical scavenging capacity (Kim & Choe, 2012).

### 3.6 Harmful substances

**Fig. 5:**
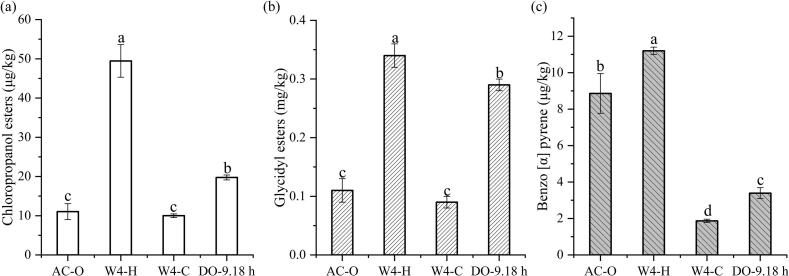
Trichloropropanol ester content (a), glycidyl ester content (b), and benzo[α]pyrene content (c) of coconut oil and coconut diacylglycerol oil under different processes. Different letters in the data plot indicate significant differences (*P* < 0.05).

The influence of different processes on the content of 3-monochloropropanediol esters (3-MCPD esters) in different oil is shown in Fig. 5 (a). Elevated temperatures and extended reaction durations are primary factors contributing to the formation of 3-MCPD esters. The concentration of 3-MCPD esters in AC-O was measured at 11.04 μg/kg, whereas in W4—H, this value increased significantly to 49.45 μg/kg (*P* < 0.05), surpassing the levels found in other oils. This increase is likely linked to the formation of lipid chlorination precursors, such as chlorinated glycerols and phospholipids, induced by high temperatures. Conversely, W4—C maintained a low temperature, effectively controlling the 3-MCPD ester content at a reduced level of 10.01 μg/kg, thereby demonstrating the efficacy of non-thermal processing methods. Furthermore, during the enzymatic hydrolysis process, the 3-MCPD ester content in the CO-DAG group was 19.76 μg/kg. Although this level was significantly higher than that in W4—C (*P* < 0.05), it remained lower than the levels observed in the hot-pressed group, suggesting that the 3-MCPD esters produced during enzymatic hydrolysis are considerably less than those generated through high-temperature heating. During the preparation process, residual water in coconut oil carries the chlorine elements from the natural salts in coconut juice. These chlorine elements react with the glycerol, which is produced through lipase hydrolysis, leading to the formation of 3-MCPD. Notably, when stainless steel equipment is utilized in industrial settings, trace corrosion of the contact surface in an acidic reaction environment (pH 5.5–6.0) can readily catalyze chlorination side reactions. This has been verified by HPLC-MS/MS, which detected the generation of derivatives such as 2-MCPD esters (Kabutey et al., 2024). Consequently, when optimizing the enzymatic process, it is crucial to balance the yield with the potential risk of chlorination contamination. To achieve a synergistic regulation of functionality and safety, it is recommended to incorporate dechlorination adsorbents, such as diatomite-supported activated carbon for targeted adsorption, into the process.

The glycidyl ester (GE) contents of various samples are shown in Fig. 5(b). AC-O exhibited the lowest glycidyl ester (GE) content, measuring 0.11 mg/kg. In contrast, the GE content of W4—H was significantly higher at 0.34 mg/kg, with a statistically significant difference compared to AC-O (*P* < 0.05). Notably, the GE level in W4—C, despite being unrefined, was slightly lower than that of AC-O, at 0.09 mg/kg. This observation can be attributed to the cold-pressing process, which prevents the initiation of thermal oxidation chain reactions, thereby inhibiting GE formation. During the enzymatic transesterification of DAG oil, an upward trend in GE content was observed with prolonged processing time. Specifically, the GE level in CO-DAG was 0.29 mg/kg, significantly higher than that in AC-O (*P* < 0.01). The formation of GEs is primarily due to the oxidative β-fragmentation of TAG at elevated temperatures. This process involves alkoxy radicals attacking the α‑hydrogen of TAG, leading to the cleavage of the glycerol backbone and the formation of GE (Liu et al., 2019). Furthermore, the GE content in CO-DAG oil was lower than that in the hot-pressed group, likely because the transesterification reaction reduces the free fatty acid content, indirectly inhibiting the formation of glycidyl groups. Although the GE content in CO-DAG oil was significantly higher than that in the cold-pressed group and the commercial group, the content of 0.29 mg/kg ≤ 1 mg/kg (Becalski et al., 2012) complies with international standards, proving its certain safety.

The influence of different processes on the benzo(*a*)pyrene (BaP) content in CO is shown in Fig. 5 (c). Thermal exposure and reaction duration during processing are key factors for BaP accumulation. The BaP concentration in AC-O was measured at 8.86 μg/kg. The elevated temperatures associated with the hot-pressed process facilitate oil carbonization and Maillard reactions, which in turn promote the formation of polycyclic aromatic hydrocarbons. Notably, the BaP content in W4—H reached 11.2 μg/kg, surpassing the national standard limit of 10 μg/kg as specified by GB 2762–2022. Conversely, W4—C exhibited the lowest BaP concentration at 1.87 μg/kg, corroborating findings by Zachara et al. (2016) that low temperatures effectively inhibit BaP formation. The BaP concentration in the CO-DAG group was 3.39 μg/kg. Although this value was significantly higher than that of W4—C (*P* < 0.01), it remained substantially lower than that observed in the hot-pressed process. The enzymatic hydrolysis process markedly reduces BaP formation and residue through mechanisms such as maintaining a low-temperature environment, employing directional catalysis, purifying raw materials, and avoiding high-temperature side reactions, resulting in lower BaP content compared to both AC-O and hot-pressed products. This difference reflects the advantages of biocatalytic processes in food safety and green processing, especially in controlling high-temperature harmful substances. Moreover, the functional properties of CO-DAG are higher than those of cold-pressed coconut oil due to the presence of DAG, providing a theoretical basis for the intensive processing of CO.

### 3.7 *In vitro* digestion evaluation

**Fig. 6:**
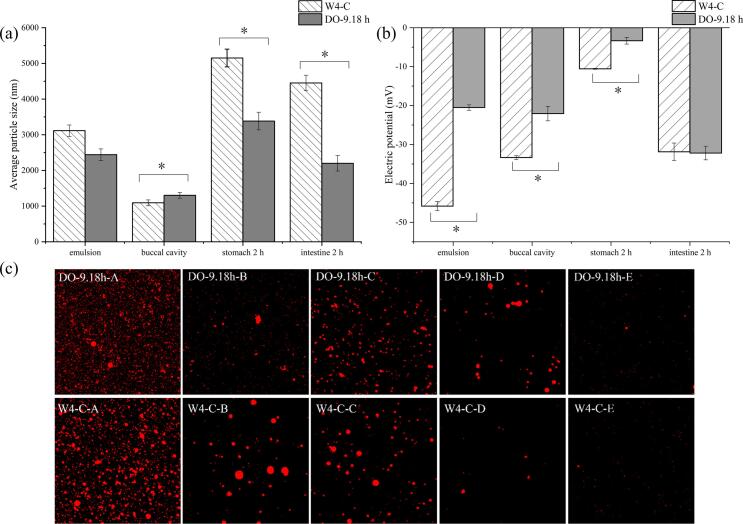
Changes in particle size (a) and electric potential (b) during different digestion stages, and microscopic images of particle morphology (c) for coconut oil and coconut diacylglycerol oil. Note: (A) Initial phase, (B) oral digestion stage, (C) gastric digestion stage, (D) intestinal digestion phase (1 h), and (E) intestinal digestion phase (2 h).

To compare the digestion characteristics between CO and CO-DAG, W4—C coconut oil and CO-DAG oil was chosen. The average particle size and zeta potential during digestion are shown in Fig. 6 (a-b). The microstructure during digestion is shown in Fig. 6(c), where A, B, C, D, and E represent the emulsion state (A), oral digestion state (B), gastric digestion state (C), intestinal digestion state at 1 h (D), and intestinal digestion state at 2 h (E) during digestion, respectively. Lipid droplets are uniformly dispersed in the emulsion without aggregation. Following oral digestion, a reduction in the average particle size of the emulsion was observed, the particle size of W4—C decreased to 1093.33 nm, whereas the CO-DAG group exhibited a larger size of 1303 nm. This observation may be attributed to more complete emulsification of DAG oil droplets under oral conditions. After 2 h of gastric digestion, the average particle size of the lipid emulsion increased significantly (*P* < 0.05). Specifically, the particle size of the W4—C group increased to 5152.33 nm, whereas the CO-DAG group experienced a more modest increase to 3384.33 nm. Microscopic observations, as depicted in Fig. 6(c), demonstrated extensive coalescence of lipid droplets during gastric digestion, however, the CO-DAG group exhibited relatively greater stability. This disparity is attributed to the synergistic effects of oil structure, particle size dynamics, and surface potential. In comparison to the structure of CO, the diester structure of CO-DAG reduces steric hindrance. Additionally, the selective catalysis of pancreatic lipase on sn-1,3 ester bonds enhances the hydrolysis rate by 15 %–25 % (Zhang et al., 2022). Furthermore, when medium-chain fatty acids, such as lauric acid, are present in the form of DAG, they can directly enter the liver for energy production following their release, exhibiting significantly greater metabolic efficiency compared to TAG. Regarding the effect of particle size, the increase in particle size of the CO-DAG group emulsion during gastric digestion was reduced by 34 %, a phenomenon attributed to the enhanced amphiphilic properties of DAG. This property facilitates greater protein adsorption onto the formed interfacial membrane, thereby increasing stability (Yang et al., 2020). During intestinal digestion, the particle size of DAG (2201 nm) is only half that of conventional coconut oil (4452 nm), resulting in a 2.3-fold increase in specific surface area. This substantial increase significantly enhances the contact efficiency with pancreatic lipase (Diao et al., 2020).

In terms of surface potential, the initial zeta potential of CO (−45.83 mV) was lower than that of CO-DAG (−20.5 mV). Following gastric digestion, the reduction in potential for CO-DAG was less pronounced (−3.33 mV compared to −10.57 mV), indicating a stronger binding affinity with proteins and a more stable interfacial membrane (Zhang et al., 2015). During intestinal digestion, the potential of both CO and CO-DAG returned to approximately −32 mV. However, CO-DAG exhibited a higher surface charge density, facilitating the adsorption of bile salts and the formation of mixed micelles with smaller particle sizes (100–200 nm), thereby enhancing lipid absorption (Chang & McClements, 2016). Furthermore, the catalytic constant of pancreatic lipase in the DAG system exceeded that in the TAG system, corroborating the notion that its charge environment enhances enzyme-substrate interactions (Rocha et al., 2024). In conclusion, CO-DAG achieves a dual improvement in digestion rate and metabolic efficiency through the optimization of fat structure (replacement of TAG with DAG), regulation of particle size (reduced particle size during intestinal digestion), and management of interfacial potential (enhanced charge stability). The *in vitro* digestion characteristics of CO-DAG exhibited a more pronounced metabolic friendliness and greater potential for health applications compared to CO.

### 3.8 FFA release and kinetic analysis during lipid digestion

**Fig. 7:**
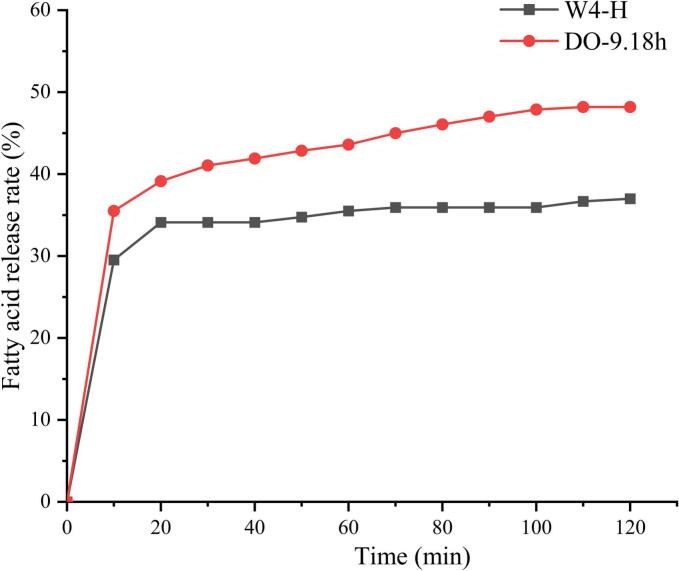
Fatty acid release rate of coconut oil and coconut diacylglycerol oil during intestinal digestion process.

The rate of fatty acid release serves as a crucial indicator for assessing the bioavailability of oils. In this study, a titration method was employed to evaluate the rate and extent of lipid digestion under simulated intestinal conditions. Fig. 7 illustrates the temporal variation in the release of FFA from lipids during intestinal digestion. The FFA release profile depicted in the figure is consistent with findings from previous studies (Pereira et al., 2022). During the initial 20 min, there is a rapid release of FFA, suggesting that pancreatic lipase promptly adheres to the surface of lipid droplets and initiates the degradation of TAG and DAG molecules. Subsequently, over an extended period, the rate of FFA release gradually increases until it reaches a relatively stable phase, culminating in the maximum level of fatty acid release (Ye et al., 2019).

Based on data from *in vitro* simulated digestion experiments, the fatty acid release profiles of W4—H and CO-DAG exhibit significant kinetic differences. Specifically, the enzymatically transesterified CO-DAG demonstrates a fatty acid release of up to 48.19 %, which is notably higher than the 36.99 % release observed in crude fatty acid oil. The precise configuration of the fatty acid release rate and time curve is influenced by factors such as the source, type, and structure of lipids within the emulsion, as well as the digestion methods employed, leading to variability in digestion levels (Ye et al., 2018). For example, pancreatic lipase specifically hydrolyzes the sn-1 and 3 fatty acyl chains of TAG. The presence of cis double bonds in unsaturated alkenyl chains poses steric hindrance, making them less susceptible to lipase-mediated hydrolysis (Zhang et al., 2022). Generally, the digestibility of fatty acids varies with different chain lengths, following the order: long-chain fatty acids < medium-chain fatty acids < short-chain fatty acids (Liang et al., 2016). In this study, CO-DAG oil is predominantly composed of short-chain saturated fatty acids, demonstrating favorable digestion characteristics.

## 4 Conclusions

This study successfully prepared CO-DAG with a high DAG content of 54.65 % through enzymatic transesterification, using Novozym 435 as the catalyst. The optimal conditions were identified as a monoglyceride-to-crude oil ratio of 1:2.5, 9.18 h reaction time, 65 °C temperature, and 3.60 % enzyme dosage, achieving a DAG content of 54.65 %. CO-DAG oil exhibited higher acid value but maintained acceptable peroxide and total oxidation values. In terms of harmful substances, the contents of benzo(*a*)pyrene, 3-monochloropropanediol esters, and glycidyl esters in all groups were within acceptable ranges, ensuring the safety of the oil products. *In vitro* digestion showed CO-DAG oil had faster particle expansion, easier lipase binding, and a 11.2 % higher fatty acid release rate than coconut oil, indicating better digestibility.

While this study successfully prepared CO-DAG, it did not conduct a detailed analysis of the glyceride composition of CO-DAG. Previous research has demonstrated that CO-DAG exhibits significantly enhanced emulsifying properties compared to traditional CO, indicating its potential applications in the food industry, cosmetics sector, and drug delivery systems. However, the investigation into the characteristics of CO-DAG remains in its nascent stages. Future research should prioritize examining the emulsification stability, antioxidant properties, bioactivity, and digestion and absorption characteristics of CO-DAG. Conducting systematic *in vitro* and *in vivo* experiments to explore its functional properties will provide a robust theoretical and practical foundation for the development of functional lipid products.

## CRediT authorship contribution statement

**Jiao-jiao Yin:** Writing – review & editing, Writing – original draft, Funding acquisition, Conceptualization. **Xin-feng Li:** Writing – original draft. **Shu Wang:** Methodology, Investigation, Formal analysis. **He Huang:** Methodology, Investigation, Formal analysis. **Zhuo-long Guan:** Methodology, Investigation, Formal analysis. **Xing-he Zhang:** Writing – review & editing. **Wu Zhong:** Writing – review & editing. **Pan Gao:** Writing – review & editing. **Dong-ping He:** Conceptualization.

## Funding

This study is supported by the 10.13039/501100012166National Key Research and Development Program of China (2023YFD2200705), and the Open Research Fund of the Key Laboratory of Edible Oil Quality and Safety, State Administration for Market Regulation (SYYKF202405).

## Declaration of competing interest

The authors declare that they have no known competing financial interests or personal relationships that could have appeared to influence the work reported in this paper.

## Data Availability

Data will be made available on request.

## Appendix A Supplementary data

Supplementary material Image 1
