# Supplementary material for: Preparation, physicochemical properties, and in vitro digestion characteristics of coconut diacylglycerol oil
Source: Food Chem X. 2025 Sep 10;31:102991. doi: 10.1016/j.fochx.2025.102991 (PMC12640045; doi:10.1016/j.fochx.2025.102991)
Supplement: Supplementary file 1 — Supplementary material [file mmc1.docx]

**Preparation, physicochemical properties, and *in vitro* digestion characteristics of coconut diacylglycerol oil**

Jiao-jiao Yin^a,b,*,1^, Xin-feng Li^a,b,1^, Shu Wang^c^, He Huang^a,b^, Zhuo-long Guan^c^, Xing-he Zhang^a,b^, Wu Zhong^a,b^, Pan Gao^a,b^, Dong-ping He^a^

^a^ Key Laboratory of Edible Oil Quality and Safety for State Market Regulation, Wuhan Polytechnic University, Wuhan 430023, China

^b^ Key Laboratory for Deep Processing of Major Grain and Oil of Ministry of Education in China, College of Food Science and Engineering, Wuhan Polytechnic University, Wuhan 430023, China

^c^ Wuhan Institute for Food and Cosmetic Control, 1137 Jinshan Avenue, Wuhan 430040, China

^1^ These authors contributed equally to this work and share first authorship

^*^Corresponding author:

Jiaojiao Yin, Ph.D

yinjiaojiao@whpu.edu.cn

Table S1 Comparison table of experimental factors and levels

| Factor level | substrate ratio（mol） | reaction time（h） | reaction temperature（℃） | the amount of enzyme added（%） |
| --- | --- | --- | --- | --- |
| -1 | 1:1 | 8 | 50 | 1 |
| 0 | 1:3 | 10 | 60 | 3.5 |
| 1 | 1:5 | 12 | 70 | 6 |

Table S2 Box-Behnken experimental scheme and results

| std | substrate ratio | reaction time | reaction temperature | the amount of enzyme added | DAG |
| --- | --- | --- | --- | --- | --- |
| 1 | -1 | -1 | 0 | 0 | 48.05 |
| 2 | 1 | -1 | 0 | 0 | 40.74 |
| 3 | -1 | 1 | 0 | 0 | 39.08 |
| 4 | 1 | 1 | 0 | 0 | 48.82 |
| 5 | 0 | 0 | -1 | -1 | 38.59 |
| 6 | 0 | 0 | 1 | -1 | 44.44 |
| 7 | 0 | 0 | -1 | 1 | 43.89 |
| 8 | 0 | 0 | 1 | 1 | 45.58 |
| 9 | -1 | 0 | 0 | -1 | 45.1 |
| 10 | 1 | 0 | 0 | -1 | 40.93 |
| 11 | -1 | 0 | 0 | 1 | 40.1 |
| 12 | 1 | 0 | 0 | 1 | 47.56 |
| 13 | 0 | -1 | -1 | 0 | 36.57 |
| 14 | 0 | 1 | -1 | 0 | 47.65 |
| 15 | 0 | -1 | 1 | 0 | 49.38 |
| 16 | 0 | 1 | 1 | 0 | 40.85 |
| 17 | -1 | 0 | -1 | 0 | 38.59 |
| 18 | 1 | 0 | -1 | 0 | 45.72 |
| 19 | -1 | 0 | 1 | 0 | 47.81 |
| 20 | 1 | 0 | 1 | 0 | 45.51 |
| 21 | 0 | -1 | 0 | -1 | 40.66 |
| 22 | 0 | 1 | 0 | -1 | 42.40 |
| 23 | 0 | -1 | 0 | 1 | 46.11 |
| 24 | 0 | 1 | 0 | 1 | 42.45 |
| 25 | 0 | 0 | 0 | 0 | 53.33 |
| 26 | 0 | 0 | 0 | 0 | 52.32 |
| 27 | 0 | 0 | 0 | 0 | 52.62 |
| 28 | 0 | 0 | 0 | 0 | 51.82 |
| 29 | 0 | 0 | 0 | 0 | 52.48 |

Table S3 ANOVA of the regression equation

| Source | Sum of Squares | df | Mean Square | F Value | p-value Prob > F |  |
| --- | --- | --- | --- | --- | --- | --- |
|  |  |  |  |  |  |  |
| Model | 638.5407 | 14 | 45.61005 | 48.48929 | < 0.0001 | significant |
| A- substrate ratio | 9.275208 | 1 | 9.275208 | 9.860728 | 0.0072 |  |
| B- reaction time | 0.005633 | 1 | 0.005633 | 0.005989 | 0.9394 |  |
| C- reaction temperature | 42.4128 | 1 | 42.4128 | 45.09021 | < 0.0001 |  |
| D- the amount of enzyme added | 15.34541 | 1 | 15.34541 | 16.31412 | 0.0012 |  |
| AB | 72.67563 | 1 | 72.67563 | 77.26345 | < 0.0001 |  |
| AC | 22.23123 | 1 | 22.23123 | 23.63462 | 0.0003 |  |
| AD | 33.81423 | 1 | 33.81423 | 35.94883 | < 0.0001 |  |
| BC | 96.13803 | 1 | 96.13803 | 102.207 | < 0.0001 |  |
| BD | 7.29 | 1 | 7.29 | 7.750198 | 0.0146 |  |
| CD | 4.3264 | 1 | 4.3264 | 4.599514 | 0.0500 |  |
| A^2 | 96.80887 | 1 | 96.80887 | 102.9202 | < 0.0001 |  |
| B^2 | 132.4922 | 1 | 132.4922 | 140.8561 | < 0.0001 |  |
| C^2 | 119.4893 | 1 | 119.4893 | 127.0323 | < 0.0001 |  |
| D^2 | 171.2537 | 1 | 171.2537 | 182.0645 | < 0.0001 |  |
| Residual | 13.1687 | 14 | 0.940621 |  |  |  |
| Lack of Fit | 7.951175 | 10 | 0.795118 | 0.609575 | 0.7608 | not significant |
| Pure Error | 5.21752 | 4 | 1.30438 |  |  |  |
| Cor Total | 651.7094 | 28 |  |  |  |  |


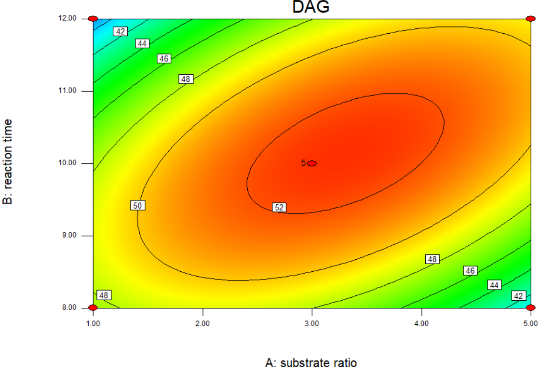

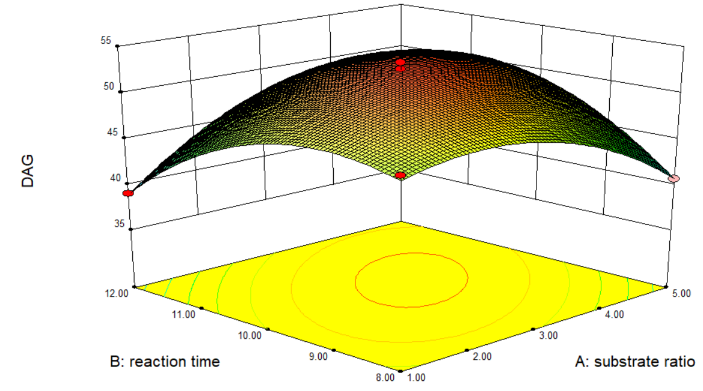


Figure S1 (a) Response surface and contour lines for the interaction between substrate ratio and reaction time


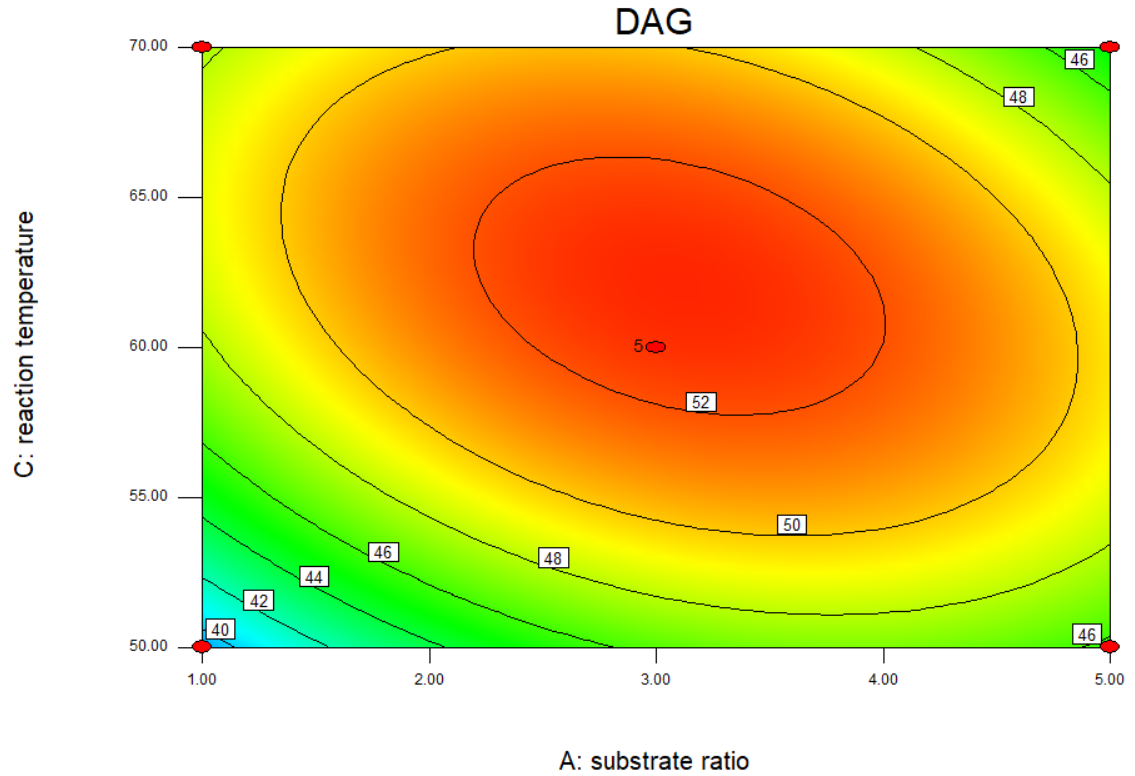

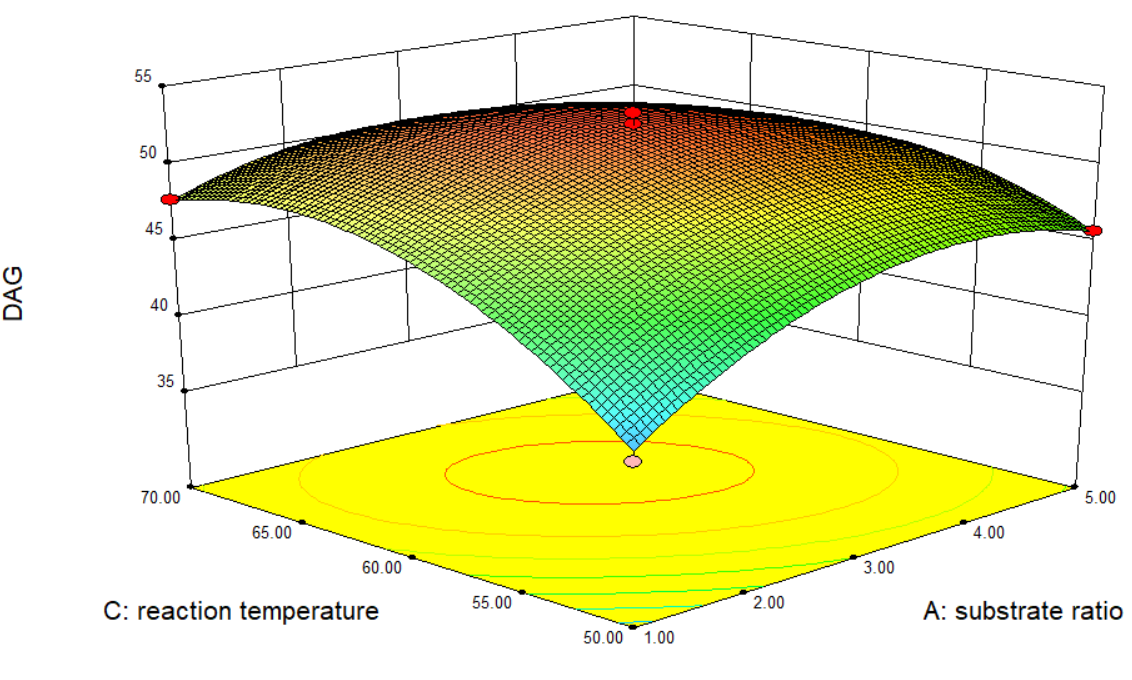


Figure S1 (b) Response surface and contour lines for the interaction between Y substrate ratio and reaction temperature


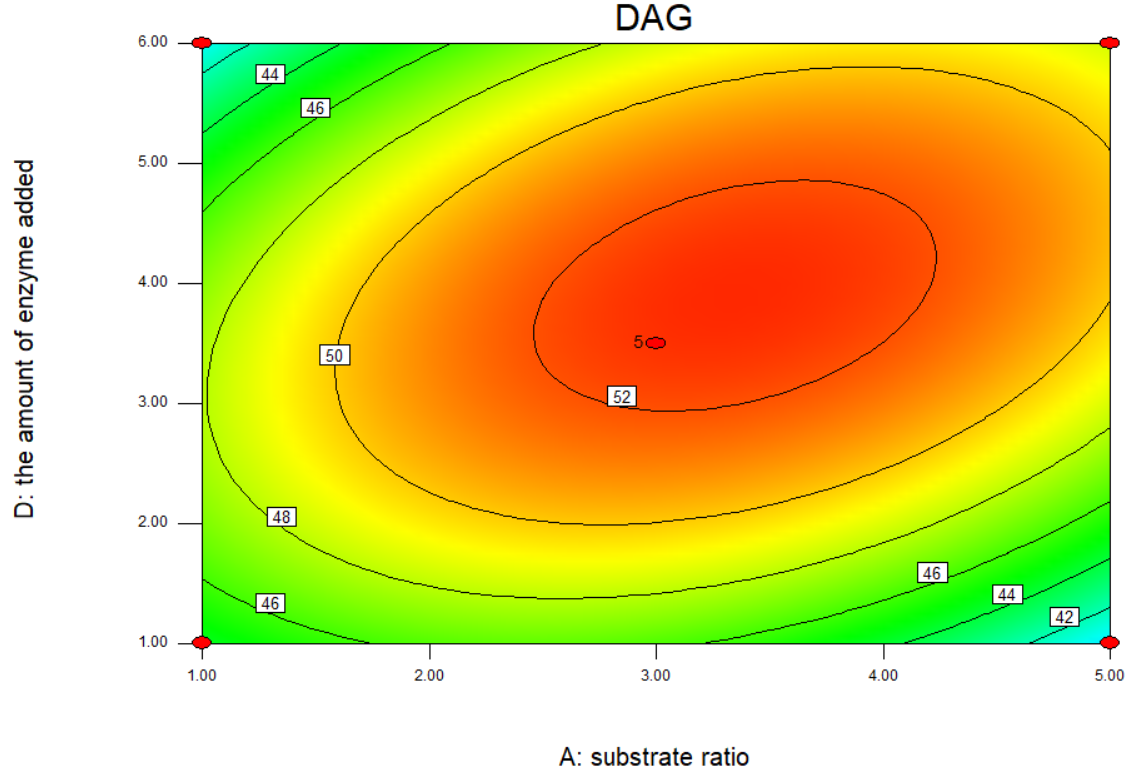

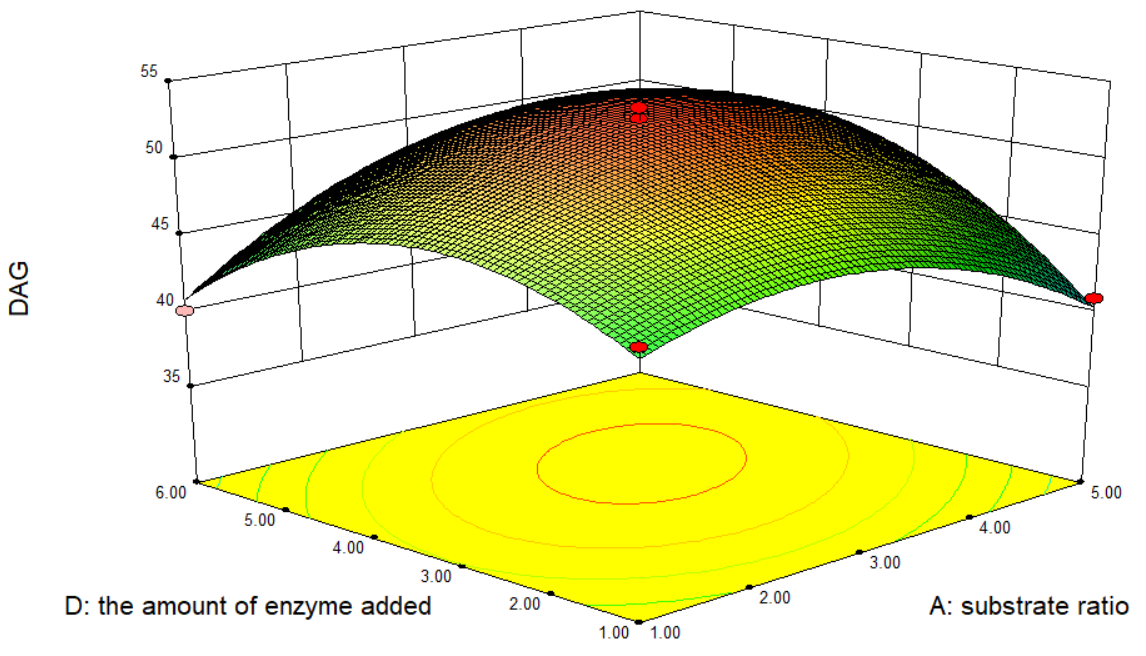


Figure S1 (c) Response surfaces and contours of the interaction between Y substrate ratio and amount of enzyme added


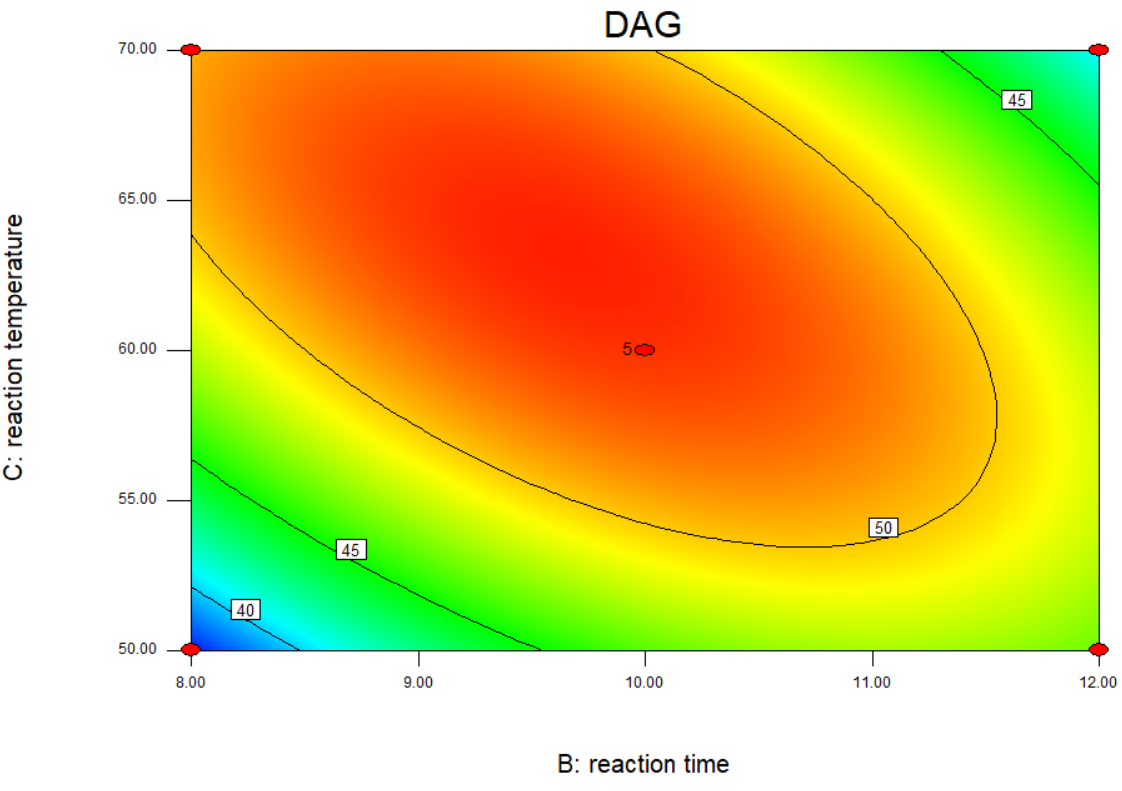

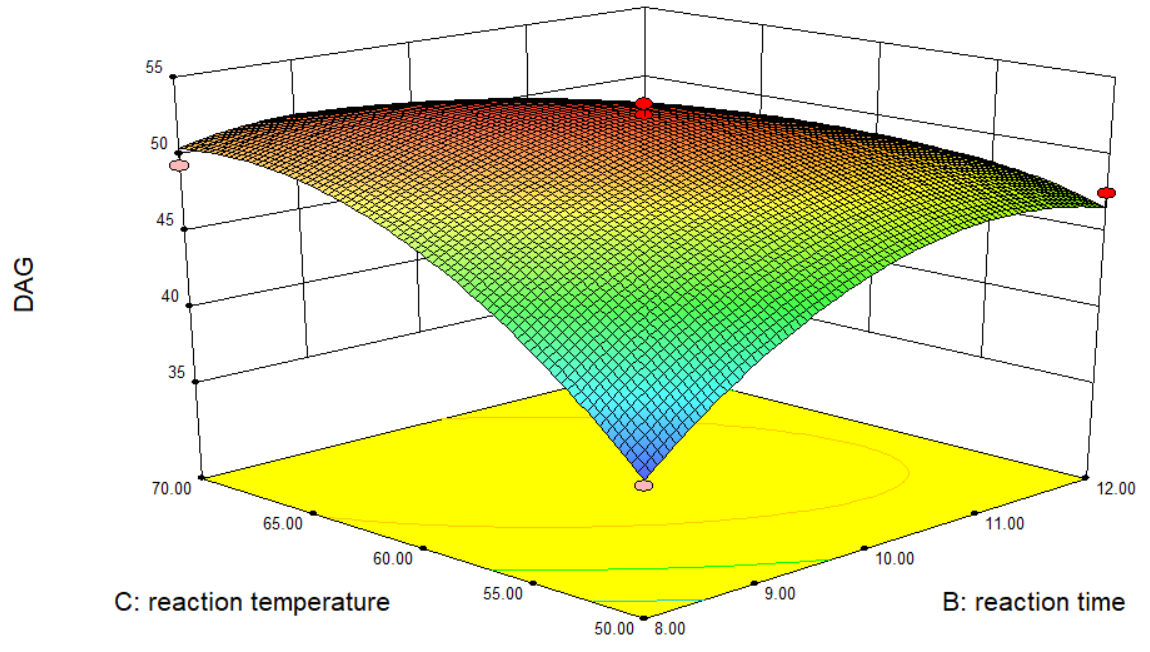


Figure S1 (d) Response surface and contour lines for the interaction between reaction time and reaction temperature


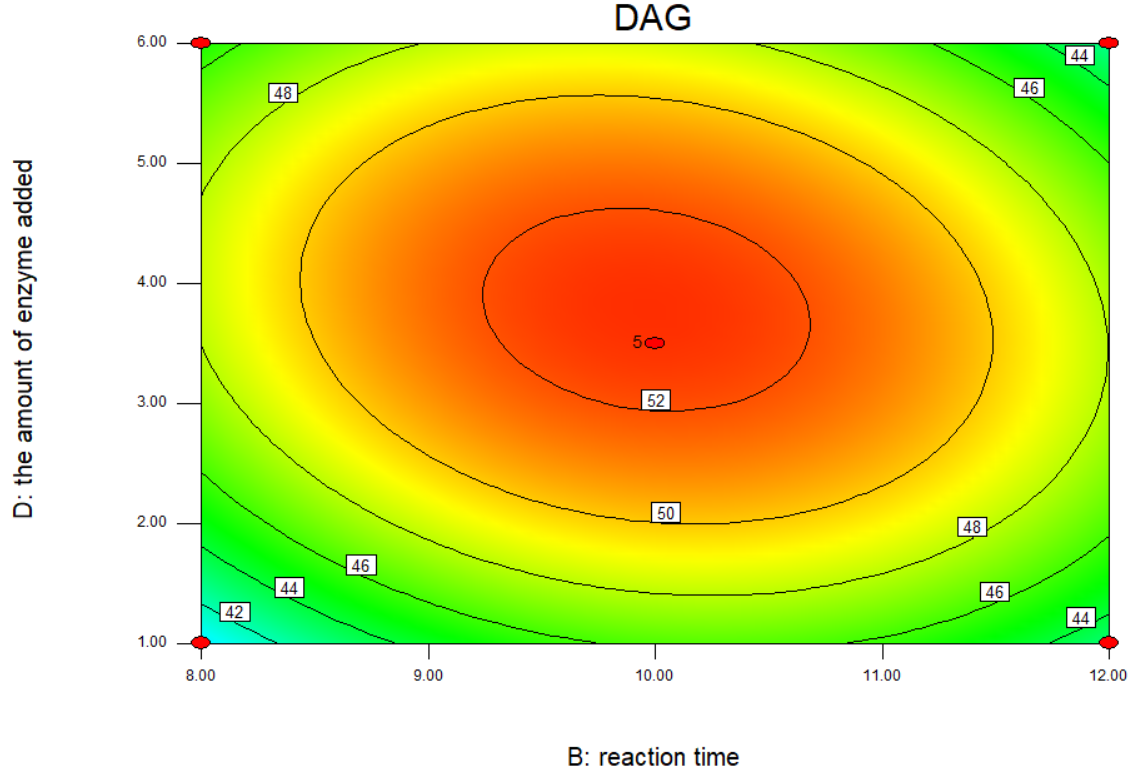

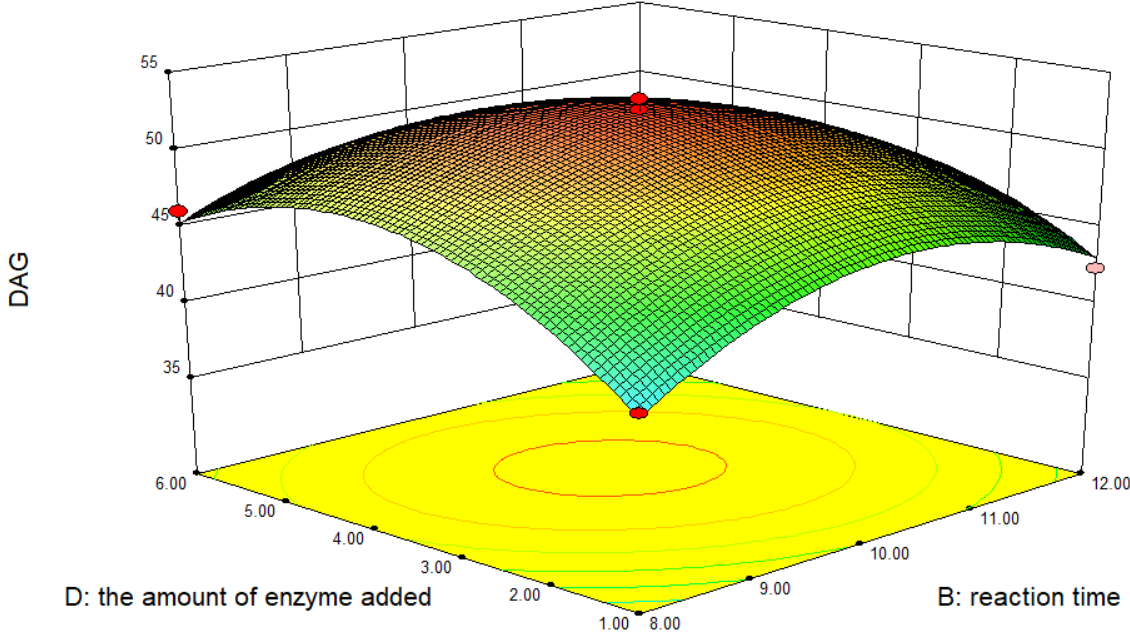


Figure S1 (e) Response surface and contour lines for the interaction between reaction time and enzyme amount added


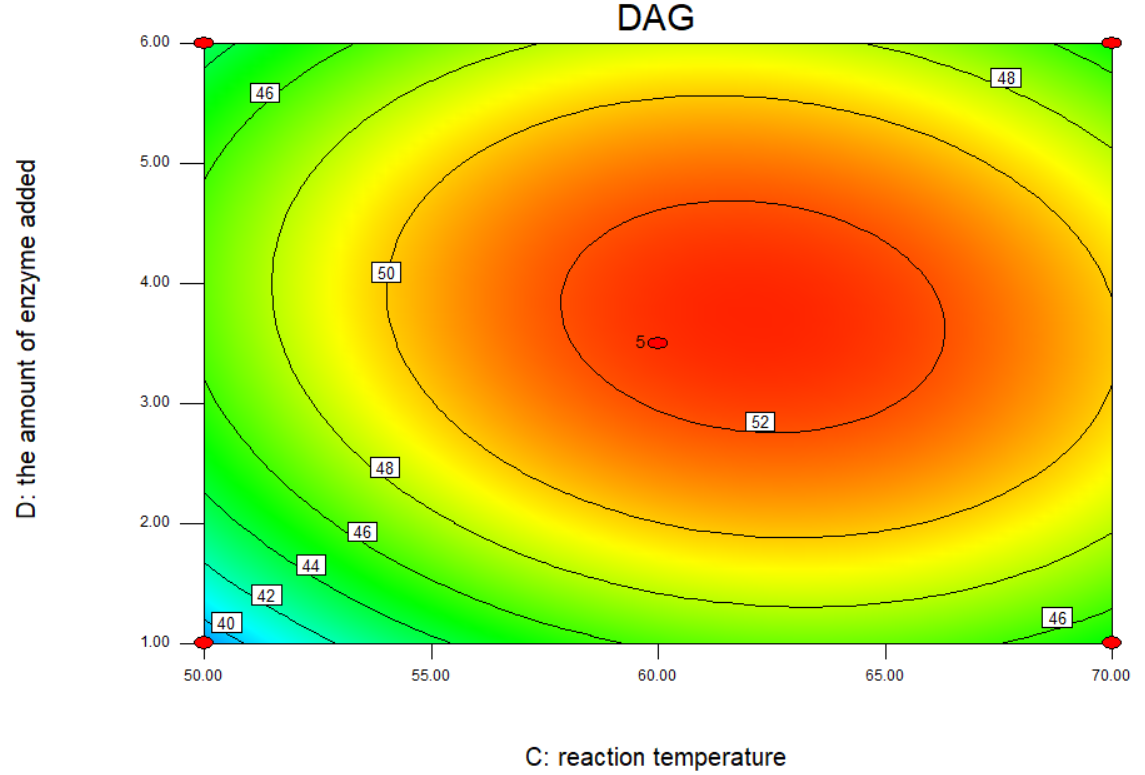

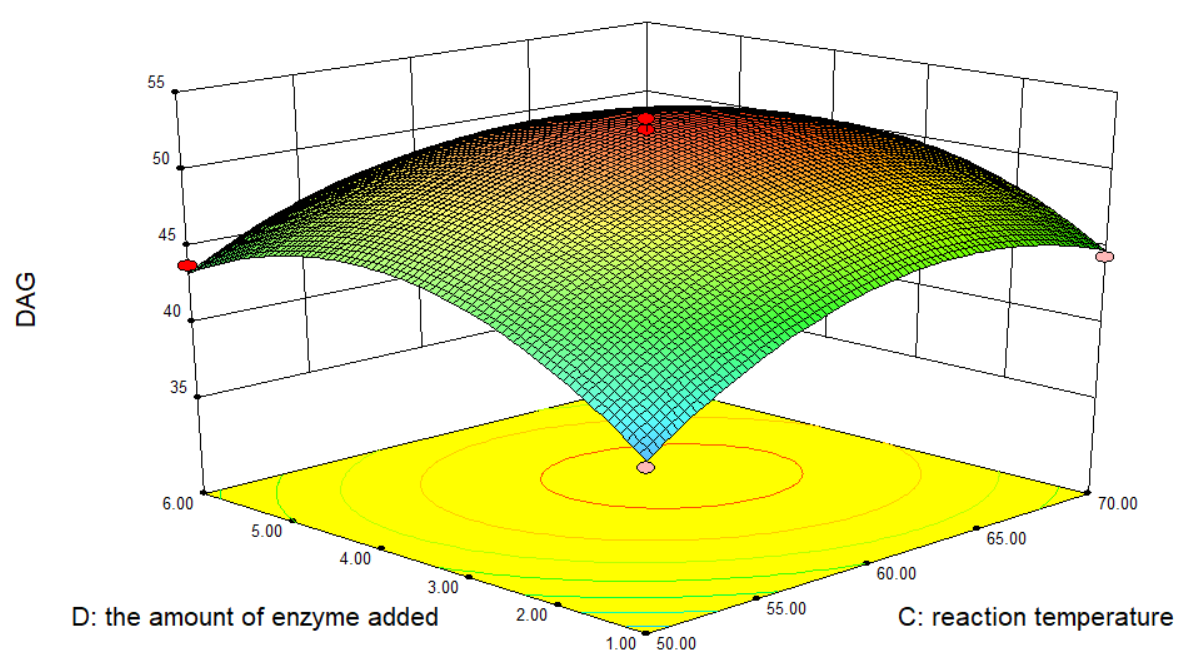


Figure S1 (f) Response surface and contour lines for interaction between reaction temperature and amount of enzyme added
